# Mental Health Service Use in Sexually and Gender Diverse Youths and Cisgender Heterosexual Youths

**DOI:** 10.1001/jamanetworkopen.2026.26797

**Published:** 2026-08-14

**Authors:** Lauren Anzarouth, Sasha MacNeil, Natalie Castellanos Ryan, Alicia Martineau, Massimiliano Orri, Caroline Temcheff, Marc Dorais, Alain Girard, Srividya N. Iyer, Ian Colman, Isabelle Ouellet-Morin, Nicholas Chadi, Robert-Paul Juster, Marie-Claude Geoffroy

**Affiliations:** 1 Department of Psychiatry, Douglas Mental Health University Institute, McGill University, Montreal, Quebec, Canada; 2 School of Psychoeducation, University of Montreal, Montreal, Quebec, Canada; 3 Department of Psychiatry, Douglas Mental Health University Institute, McGill University, Montreal, Quebec, Canada; 4 Department of Epidemiology, Biostatistics and Occupational Health, McGill University, Montreal, Quebec, Canada; 5 Danish Research Institute for Suicide Prevention, Mental Health Centre Copenhagen, Copenhagen, Denmark; 6 Department of Educational and Counselling Psychology, McGill University, Montreal, Quebec, Canada; 7 StatSciences Inc, Notre-Dame-de-l’Île-Perrot, Quebec, Canada; 8 Research Unit on Children’s Psychosocial Maladjustment, University of Montreal, Montreal, Quebec, Canada; 9 School of Epidemiology and Public Health, University of Ottawa, Ottawa, Ontario, Canada; 10 School of Criminology, University of Montreal, Montreal, Quebec, Canada; 11 Centre Hospitalier Universitaire Sainte-Justine Research Centre, Department of Pediatrics, University of Montreal, Montreal, Quebec, Canada; 12 Department of Psychiatry and Addiction, University of Montreal, Montreal, Quebec, Canada

## Abstract

**Importance:**

Sexually and gender diverse (SGD) youths experience disproportionate mental health challenges, yet longitudinal evidence of their mental health service use remains limited.

**Objective:**

To examine mental health outpatient, emergency, and inpatient service use among SGD youths compared with cisgender heterosexual youths from preschool to young adulthood.

**Design, Setting, and Participants:**

This population-based cohort study included 1326 participants from the Quebec Longitudinal Study of Child Development who self-reported sexual orientation and gender identity at age 23 years. Participants were followed up from birth (between October 1997 and July 1998). The wave of data collection when they were 23 years old occurred in June 2023. Data analyses were conducted between September 2024 and June 2026.

**Exposure:**

Self-reported sexual and gender diversity at age 23 years.

**Main Outcomes and Measures:**

Mental health outpatient (age 1-23 years; 1997-1998 to 2021), emergency (age 18-23 years; 2016 to 2021), and inpatient (age 8-23 years; 2006 to 2021) service use extracted from 3 provincial administrative health databases: the Régie de l’Assurance Maladie du Québec, the Banque de Données Communes des Urgences, and the Maintenance et Exploitation des Données pour l’Étude de la Clientèle Hospitalière.

**Results:**

Of 1326 participants (691 [52.1%] assigned female at birth), 324 (24.4%) identified as sexually diverse and 32 (2.4%) identified as gender diverse. After controlling for sex assigned at birth and socioeconomic status, SGD youths were more likely than cisgender heterosexual youths to use outpatient services (adjusted odds ratio [AOR], 1.41 [95% CI, 1.09-1.83]) and emergency services (AOR, 1.99 [95% CI, 1.33-2.98]); the association was not present for inpatient services (AOR, 1.49 [95% CI, 0.87-2.54]). Growth curve models indicated a similar likelihood of outpatient service use between SGD youths and cisgender heterosexual youths in preschool, but steeper increases among SGD youths over time (*b* = 0.21 [SE, 0.11; 95% CI, −0.002 to 0.41]; *P* = .05]). SGD youths assigned female at birth had higher odds of emergency and inpatient service use compared with cisgender heterosexual females. SGD youths assigned male at birth had higher odds of outpatient service use compared with cisgender heterosexual males.

**Conclusions and Relevance:**

In this population-based cohort study, SGD youths showed greater use of mental health services across development, with disparities emerging early and widening over time. These findings highlight the need for early and sustained mental health support for SGD youths.

## Key Points

**Question:**

What differences exist in mental health service use between sexually and gender diverse (SGD) youths and their cisgender heterosexual peers across development?

**Findings:**

In this cohort study of 1326 youths in Quebec, Canada, linked to administrative health records, SGD youths were more likely to use outpatient and emergency mental health services than their cisgender heterosexual peers and had steeper increases in outpatient service use over time.

**Meaning:**

These findings suggest the need for early, tailored, and affirming mental health care for SGD youths.

## Teaser

This cohort study examines differences in mental health service use among sexually and gender diverse youths compared with their cisgender heterosexual peers across outpatient, emergency, and inpatient services.

## Introduction

Sexually and gender diverse (SGD) youths (eg, lesbian, gay, bisexual, transgender, nonbinary, or questioning) represent a growing population, accounting for the largest proportion of communities consisting of lesbian, gay, bisexual, transgender, queer, intersex, 2-spirit, and additional persons who identify as SGD in North America.^1,2^ Despite increasing sociopolitical recognition in Canada and globally,^3,4^ SGD youths continue to face discrimination^5,6^ and report significantly poorer mental health than their cisgender heterosexual peers.^7^ Findings from cross-sectional and longitudinal studies, including those from the Quebec Longitudinal Study of Child Development,^8,9^ consistently show that SGD youths report more mental health problems, including higher rates of anxiety, depression, substance use, and suicidality, than cisgender heterosexual youth.^7,8,9,10,11,12,13^ Bisexual youths appear to be particularly vulnerable,^13,14,15^ likely reflecting the unique stigmatization they face from both heterosexual and lesbian and gay communities.^16^

Emerging research shows that SGD individuals access mental health services at higher rates than their cisgender heterosexual peers.^17,18,19^ A national cross-sectional survey of 191 954 US adults found higher self-reported mental health service use among sexually diverse individuals, compared with their heterosexual counterparts,^17^ and comparable disparities were observed in a sample of 33 220 US college students.^18^ However, these studies relied on self-reported data, which are prone to recall and social desirability biases.^20,21^ Accordingly, recent research has begun to incorporate administrative health records to capture mental health service use,^15,22,23^ with studies among transgender veterans in the US^23^ and transgender individuals^22^ and sexually diverse adults in Canada^15^ each reporting elevated mental health outpatient (ie, general practitioner visit), inpatient (ie, hospitalizations), and emergency service use compared with cisgender heterosexual individuals.

To our knowledge, existing research has primarily focused on adults, leaving mental health service use earlier in life largely unexplored. This gap is important because disparities in mental health symptoms often emerge in early adolescence, a key period for sexual and gender identity formation.^24,25,26,27^ Understanding when SGD youths first seek services is essential to inform prevention efforts and service planning, while examining disparities in inpatient and emergency service use can provide insight into symptom severity, potential gaps in care, and unmet mental health needs.

Using administrative health records linked to a contemporary, population-based cohort from the province of Quebec, Canada, the goal of this study was to examine differences between SGD youths and cisgender heterosexual youths in mental health service use across 3 settings: outpatient (age 1-23 years), emergency (age 18-23 years), and inpatient (age 8-23 years) services, based on database coverage. To assess developmental differences, we examine outpatient trajectories across 4 periods: preschool (age 1-5 years), childhood (age 6-12 years), adolescence (age 13-17 years), and young adulthood (age 18-23 years).^28^ Differences are also explored by sex assigned at birth.

## Methods

### Participants

Participants in this cohort study were drawn from the Quebec Longitudinal Study of Child Development,^29^ a prospective birth cohort managed by the Institut de la Statistique du Québec (ISQ). The cohort originally included 2120 individuals born between October 1997 and July 1998, in the Canadian province of Quebec. The wave of data collection when they were 23 years old occurred in June 2023. Participants were recruited from provincial birth registries and were broadly representative of the sociodemographic characteristics of children born in Quebec in 1997 and 1998. At 5 months of age, race and ethnicity were parent reported using predefined categories; approximately 90% were parent-reported as White, while 11 other racial and ethnic groups (including Arab or West Asian, Black, Chinese, Filipino, Japanese, Korean, Latin American, Native or Aboriginal, South Asian, South East Asian, and other) were represented in numbers too small to report separately. Extremely preterm or postterm births, children whose mothers did not speak French or English, and those from Cree, Inuit, or other Indigenous communities were excluded due to differences in sociocultural context and institutional services. Anonymous administrative health record linkage was completed for 2082 participants (98.1% of the original sample). The final analytic sample included 1326 participants who provided sexual orientation and gender identity information at age 23 years. A total of 794 of 2082 participants (38.1%) did not provide this information and were therefore excluded. Compared with those excluded, included participants were more likely to be female (764 of 1326 [57.6%] vs 276 of 794 [34.8%]) and White (1216 of 1323 [91.9%] vs 682 of 794 [85.9%]) and to come from 2-parent households (1235 of 1323 [93.3%] vs 706 of 789 [89.5%]) with sufficient income (1069 of 1311 [81.5%] vs 529 of 770 [68.7%]) (eTable 1 in Supplement 1).

Ethical approval was obtained from ISQ, the Centre Hospitalier Universitaire Sainte-Justine, the Montreal West Island Integrated University Health and Social Services Centre, and McGill University. Written informed consent or assent was obtained from participants and/or their parents throughout. More details on the cohort are available at the web page for the Quebec Longitudinal Study of Child Development^29^ and the cohort profile.^30^ We followed the Strengthening the Reporting of Observational Studies in Epidemiology (STROBE) and the Reporting of Studies Conducted Using Observational Routinely-Collected Health Data (RECORD) reporting guideline.

### Measures

#### Sexual and Gender Diversity

Sexual orientation was self-reported at age 23 years using an adapted Klein Sexual Orientation Grid item.^31^ Participants selected 1 of 9 response options, ranging from exclusively heterosexual to exclusively homosexual, with additional options for bisexual, asexual, and questioning or unsure identities (eTable 2 in Supplement 1). Participants endorsing any identity other than exclusively heterosexual were classified as sexually diverse, consistent with a continuum-based conceptualization of sexual orientation in which varying degrees of same-sex attraction fall within a broader spectrum of sexual diversity^32,33,34^ and evidence that individuals identifying as mostly heterosexual show mental health outcomes comparable to those identifying as lesbian, gay, or bisexual.^35,36^ Gender identity was assessed by comparing participants’ sex assigned at birth (ie, assigned female or male) drawn from their birth registries in 1997 or 1998, with their self-identified gender at age 23 years in 2021. Gender identity response options included male, female, or free-text “please specify” (including nonbinary identities, eg, agender, bigender, 2-spirit, gender-fluid). Participants were classified as cisgender when their gender identity matched their sex assigned at birth and as gender diverse when it differed, following the gold standard approach in medical research.^13,37,38,39^ Participants identifying as cisgender heterosexual constituted the reference group. All other participants were classified as SGD.

#### Mental Health Service Use

Mental health service use was defined as any recorded contact associated with a mental health disorder (ie, relevant *International Classification of Diseases, Ninth Revision*, or *International Statistical Classification of Diseases, Tenth Revision*, or reason-for-visit codes) across 3 provincial databases: outpatient physician claims from the Régie de l’Assurance Maladie du Québec, emergency department visits from the Banque de Données Communes des Urgences, and inpatient discharge records from the Maintenance et Exploitation des Données pour l’Étude de la Clientèle Hospitalière (eTable 3 in Supplement 1). In Québec, minors 14 years or older may consent independently to health care services, whereas younger minors require parental or guardian consent.^40^ Outpatient service use was examined across 4 periods, reflecting key developmental stages^28,41,42^: preschool (age 1-5 years), childhood (age 6-12 years), adolescence (age 13-17 years), and young adulthood (age 18-23 years). Based on database coverage,^43^ emergency service use was examined for age 18 to 23 years (2016-2021), and inpatient service use was examined for age 8 to 23 years (2006-2021) (eTable 3 in Supplement 1). Each outcome was dichotomized as any use (≥1) and no use (0).

#### Covariates

Sex assigned at birth was categorized as assigned female or male. Socioeconomic status (SES)—a composite of income, parental educational level, and occupational prestige^44^—was reported by parents at 5 months and included in analyses due to prior associations between family SES and mental health service use.^45,46^

### Statistical Analysis

Data were analyzed between September 2024 and June 2026. Statistical analyses were conducted using SPSS Statistics, version 28 (IBM Corp) and Mplus, version 8.11 (Muthén & Muthén). Statistical significance was evaluated using 2-sided tests with a threshold of *P* < .05. Early life characteristics were compared between SGD and cisgender heterosexual youths. Effect sizes were reported as Cramer *V* for categorical variables and absolute Spearman correlations for continuous variables.

Descriptive analyses characterized the sample and estimated prevalence of service use by care setting and diagnostic category. Logistic regressions examined differences between SGD and cisgender heterosexual youths in (1) any outpatient service use from age 1 to 23 years, (2) any emergency service use from age 18 to 23 years, and (3) inpatient service use from age 8 to 23 years, with statistical significance assessed using Wald tests. Results are presented as odds ratios (ORs) with 95% CIs.

Growth curve modeling^47^ estimated trajectories of the likelihood of outpatient service use from age 1 to 23 years across 4 developmental periods: preschool (age 1-5 years), childhood (age 6-12 years), adolescence (age 13-17 years), and young adulthood (age 18-23 years). Estimated latent variables included the intercept, representing baseline likelihood of outpatient service use in preschool, and the slope, representing linear change in likelihood across all developmental periods. An unconditional model was initially fitted to examine overall linear trajectories of outpatient service use over time. Subsequent analyses tested the effect of SGD status on the intercept and the slope, with a dummy-coded variable comparing SGD youths (coded as 1) and cisgender heterosexual youths (coded as 0 [reference group]). Statistical inference for growth curve parameters (ie, SGD effects on the intercept and slope) was based on *z* tests (*z* = estimate/SE). Final models were estimated using maximum likelihood with robust standard errors (MLR) with a logit link, which appropriately handles binary outcomes and provides a likelihood-based χ^2^ test of model fit, but does not provide conventional fit indices (eg, comparative fit index, Tucker-Lewis index, root mean square error of approximation, standardized root mean square residual). As a sensitivity analysis, models were also estimated using the weighted least squares mean- and variance-adjusted estimator.^48^ Logistic regressions at each developmental period were conducted to assess whether group differences observed in outpatient service use were consistent with those identified in the growth model.

Survey weights provided by ISQ were used in all analyses to adjust for selective attrition. All models were adjusted for family SES at 5 months, with sex assigned at birth examined as both a covariate and stratification variable to assess sex-specific effects.^49^

## Results

Of the 1326 participants (691 [52.1%] assigned female and 635 [47.9%] assigned male; 1169 [88.2%] White and 153 [11.5%] racial or ethnic minority group), 340 (25.6%) were SGD and 986 (74.4%) were cisgender heterosexual. Compared with cisgender heterosexual youths, SGD youths were more likely to be assigned female at birth (248 [72.9%] vs 443 [44.9%]; Cramer *V* = 0.25) and slightly less likely to be members of a racial or minority group (24 [7.0%] compared with 129 [13.1%]; Cramer *V* = 0.08). The groups were otherwise broadly comparable in early life characteristics (Table 1).^44,50,51^ Among the 340 SGD youths, 324 (95.0%) identified as sexually diverse, most commonly as mostly heterosexual (156 [45.9%]) or bisexual (85 [25.0%]). Additionally, 32 of 340 SGD youths (9.4%) identified as gender diverse (eTable 4 in Supplement 1).

**Table 1.** Comparison of Early-Life Characteristics Between SGD and Cisgender Heterosexual Youths^a^

Characteristic	Participant group		Effect size	*P* value
	SGD (n = 340)	Cisgender heterosexual (n = 986)		
Sex assigned at birth, No. (%)				
Female	248 (72.9)	443 (44.9)	Cramer *V*, 0.25	<.001
Male	92 (27.1)	543 (55.1)		
Race and ethnicity, No. (%)				
Minority group^b^	24 (7.0)	129 (13.1)	Cramer *V*, 0.08	.002
White	317 (93.0)	852 (86.9)		
Single parent household, No. (%)				
Yes	27 (7.9)	89 (9.1)	Cramer *V*, 0.02	.52
No	314 (92.1)	893 (90.9)		
Socioeconomic status, mean (SD)^c^	0.07 (1.02)	−0.01 (1.001)	Spearman correlation, 0.04	.17
Maternal age at child’s birth, mean (SD), y	29.12 (5.40)	29.02 (5.19)	Spearman correlation, 0.0005	.99
Maternal depressive symptoms, mean (SD)^d^	1.46 (1.30)	1.51 (1.45)	Spearman correlation, 0.01	.81
Internalizing symptoms^e^	0.99 (1.25)	0.88 (1.20)	Spearman correlation, 0.04	.14
Externalizing symptoms^e^	5.20 (3.07)	5.06 (2.90)	Spearman correlation, 0.0005	.99

Abbreviation: SGD, sexually and gender diverse.

^a^ Data are compiled from the Québec Longitudinal Study of Child Development (1998-2021).^29^ All measures were reported by parents when children were 5 months of age, unless otherwise indicated. Data were weighted to adjust for selective attrition. Sample sizes may not sum to group totals due to missing data for race and single-parent household variables.

^b^ Includes parental identification of the children as Arab or West Asian, Black, Chinese, Filipino, Japanese, Korean, Latin American, Native or Aboriginal, South Asian, South East Asian, or other race or ethnicity.

^c^ Standardized (*z* score) composite index based on parental education, occupational prestige, and annual household income measured at age 5 months. Higher scores indicate higher socioeconomic status.^44^

^d^ Based on the 12-item version of the Center for Epidemiologic Studies–Depression scale, rescaled to range from 0 to 10, with higher scores indicating more depressive symptoms.^50^

^e^ Based on the Behavior Questionnaire scores (range, 1-3) of 6 items for internalizing and 10 items for externalizing behaviors, with higher scores indicating more internalizing or externalizing symptoms.^51^ Measured when children were 29 months of age; missing values were replaced with data collected at 17 months.

Outpatient mental health services were accessed by 203 of 340 SGD youths (59.7%) compared with 504 of 986 cisgender heterosexual youths (51.1%) across all 4 developmental periods, including 29 (8.5%) vs 121 (12.3%) in preschool, 83 (24.4%) vs 209 (21.2%) in childhood, 96 (28.2%) vs 231 (23.4%) in adolescence, and 139 (40.9%) vs 302 (30.6%) in young adulthood. Emergency department visits were accessed by 51 SGD youths (15.0%) vs 75 cisgender heterosexual youths (7.6%) in young adulthood (age 18-23 years). Inpatient services were accessed by 25 SGD youths (7.4%) vs 47 cisgender heterosexual youths (4.8%) aged 8 to 23 years (eTable 5 in Supplement 1). Common diagnoses among the sample included mental disorders in childhood or anxiety-related disorders for outpatient services, anxiety or situational crises for emergency services, and neurotic, stress-related, and somatoform disorders for inpatient services (eTables 6-8 in Supplement 1).

**Figure 1.**
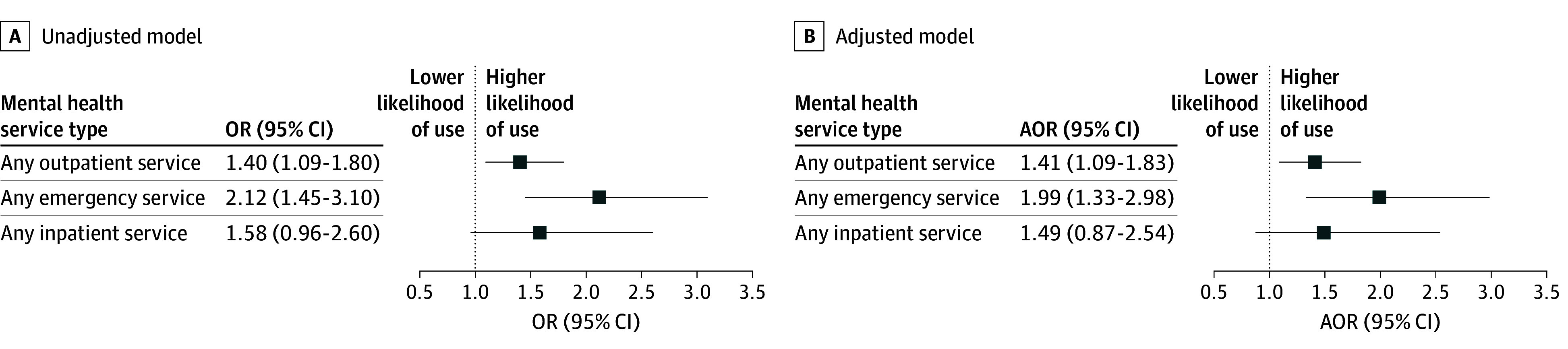
Forest Plots Showing Unadjusted and Sex- and Socioeconomic Status (SES)–Adjusted Associations of Sexual and Gender Diversity With Use of Mental Health Services Includes 1326 participants; data were weighted to adjust for selective attrition. Estimates are based on odds ratios (ORs) from logistic regressions with 95% CIs (error bars). Cisgender heterosexual youths comprised the reference group. AOR indicates adjusted OR.

After adjusting for sex and SES, SGD youths were more likely to use outpatient services (adjusted OR [AOR], 1.41 [95% CI, 1.09-1.83]) and emergency services (AOR, 1.99 [95% CI, 1.33-2.98]). Although a higher proportion of SGD youths used inpatient services compared with cisgender heterosexual youths (25 [7.4%] vs 47 [4.8%]), an association was not present (AOR, 1.49 [95% CI, 0.87-2.54]). CIs for inpatient services were wide, possibly due to the small number of users (Figure 1).

Outpatient service use increased linearly over time, with significant variability in both the intercept (σ^2^ = 2.5 [SE = 0.7]) (*P* < .001) and slope (σ^2^ = 0.5 [SE = 0.1]) (*P* < .001), indicating heterogeneity across individuals. Although the unconditional MLR model showed poor global fit (χ^2^_10_ = 50.16) (*P* < .001), this index alone is highly sensitive to sample size. Weighted least squares mean- and variance-adjusted estimates demonstrated excellent fit and produced estimates comparable to those using MLR (eTables 9 and 10 in Supplement 1) but did not converge when sex assigned at birth was included, likely due to the smaller number of SGD youths who were assigned male. Final models were therefore estimated using MLR.

**Figure 2.**
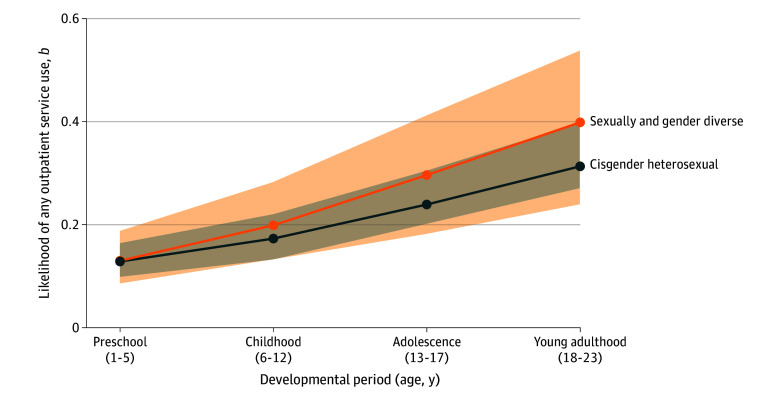
Line Graph Showing Sex- and Socioeconomic Status (SES)–Adjusted Trajectories of Use of Outpatient Mental Health Services by Sexual and Gender Diversity Includes 1326 participants; data were weighted to adjust for selective attrition. Data were compiled from the Québec Longitudinal Study of Child Development (1998-2021).^29^ Shaded areas indicate 95% CIs.

Sex- and SES-adjusted growth models indicated no intercept difference between SGD youths and their cisgender heterosexual peers in outpatient service use during preschool (*b* = 0.02 [SE, 0.22; 95% CI, −0.42 to 0.45]) (*P* = .94). Both groups showed increasing trajectories over time, with SGD youths demonstrating a significantly steeper increase in the likelihood of outpatient service use relative to their cisgender heterosexual peers (*b* = 0.21 [SE, 0.11; 95% CI, −0.002 to 0.41]) (*P* = .05) (Table 2 and Figure 2). Logistic regressions at each developmental period yielded similar results (eFigure 1 in Supplement 1).

**Table 2.** Growth Curve Modeling of Mental Health Service Use From 5 Months to 23 Years of Age Among SGD and Cisgender Heterosexual Youths With MLR Estimation^a^

Model	Intercept		Slope	
	*b* (SE) [95% CI]	*P* value	*b* (SE) [95% CI]	*P* value
Unconditional growth model (n = 1326)	[Reference]	NA	0.46 (0.07) [0.32 to 0.61]	<.001
SGD vs cisgender heterosexual, unadjusted (n = 1326)	−0.26 (0.22) [−0.69 to 0.16]	.23	0.35 (0.10) [0.14 to 0.55]	<.001
SGD	−0.26 (0.22) [−0.69 to 0.16]	.23	0.72 (0.10) [0.52 to 0.92]	<.001
Cisgender heterosexual	[Reference]	NA	0.37 (0.08) [0.22 to 0.53]	<.001
SGD vs cisgender heterosexual, adjusted for SES (n = 1320)	−0.22 (0.22) [−0.65 to 0.20]	.30	0.33 (0.10) [0.13 to 0.53]	<.001
SGD	0.23 (0.22) [−0.65 to 0.20]	.30	0.70 (0.10) [0.50 to 0.90]	<.001
Cisgender heterosexual	−0.001 (0.001) [−0.002 to 0.001]	.40	0.37 (0.08) [0.22 to 0.52]	<.001
SGD vs cisgender heterosexual, adjusted for SES and sex (n = 1326)	0.02 (0.22) [−0.42 to 0.45]	.94	0.21 (0.11) [−0.002 to 0.41]	.05
SGD	−0.44 (0.21) [−0.85 to −0.02]	.04	0.59 (0.10) [0.39 to 0.79]	<.001
Cisgender heterosexual	−0.45 (0.10) [−0.65 to −0.26]	<.001	0.39 (0.08) [0.23 to 0.54]	<.001
SGD vs cisgender heterosexual (adjusted for SES, assigned female at birth) (n = 688)	−0.10 (0.26) [−0.61 to 0.41]	.71	0.23 (0.13) [−0.03 to 0.48]	.08
SGD	−0.10 (0.26) [−0.61 to 0.41]	.70	0.79 (0.13) [0.55 to 1.04]	<.001
Cisgender heterosexual	−0.001 (0.001) [−0.003 to 0.002]	.62	0.57 (0.11) [0.34 to 0.79]	<.001
SGD vs cisgender heterosexual (adjusted for SES, assigned male at birth) (n = 632)	0.21 (0.37) [−0.52 to 0.93]	.58	0.20 (0.18) [−0.15 to 0.55]	.26
SGD	0.20 (0.37) [−0.52 to 0.93]	.58	0.40 (0.17) [0.06 to 0.74]	.02
Cisgender heterosexual	−0.001 (0.01) [−0.003 to 0.001]	.46	0.20 (0.11) [−0.03 to 0.42]	.09

Abbreviations: MLR, maximum likelihood with robust standard errors; NA, not applicable; SES, socioeconomic status; SGD, sexual and gender diversity.

^a^ Data are compiled from the Québec Longitudinal Study of Child Development (1998-2021).^29^ Data were weighted to adjust for selective attrition. Unconditional growth intercept is fixed to zero. Estimates are presented on the logit scale, explaining the negative intercepts. Model parameters are estimated for the indicated variable (coded 1) in reference to the condition in parentheses (coded 0).

Sex-stratified, SES-adjusted analyses indicated that, compared with cisgender heterosexual females, SGD youths assigned female were approximately twice as likely to use emergency services (AOR, 2.02 [95% CI, 1.25-3.29]) and inpatient services (AOR, 1.87 [95% CI, 0.98-3.57]) but not outpatient services (eFigure 2 in Supplement 1). In contrast, SGD youths assigned male at birth were more likely than cisgender heterosexual males to use outpatient services (AOR, 1.79 [95% CI, 1.18-2.82]) (eFigure 2 in Supplement 1). Outpatient service use increased over time among both SGD and cisgender heterosexual females. SGD youths assigned female showed a tendency toward steeper increases compared with cisgender heterosexual females, although this difference was not statistically significant (*b* = 0.23 [SE, 0.13; 95% CI, −0.03 to 0.48]; *P* = .08). Among youths assigned male, outpatient service use increased significantly over time among SGD youth but not cisgender heterosexual youth; however, trajectories did not differ significantly between groups (Table 2 and eFigure 3 in Supplement 1). Emergency and inpatient service use among youths assigned male at birth were not interpreted due to small cell sizes (n ≤ 10) (eTable 11 in Supplement 1).

## Discussion

To our knowledge, this is the first study to leverage linked administrative health records from a prospective birth cohort to investigate longitudinal mental health service use among SGD youths. SGD youths were more likely than cisgender heterosexual youths to use outpatient and emergency services. When mapping developmental trajectories of outpatient service visits, no group differences were observed in preschool-aged children; however, SGD youths showed steeper increases in the likelihood of outpatient service use over time.

A growing number of studies show that SGD youths report more mental health problems than their cisgender heterosexual peers,^7,8,9,10,11,12,13^ with evidence that such disparities emerge in childhood (at age 9-11 years) for outcomes such as depression, suicidality, and parent-reported emotional and behavioral problems.^24,25^ Our findings add to the literature by showing that outpatient service use disparities follow a similar developmental pattern. No differences were observed during preschool, suggesting that disparities are not yet apparent in medical services at this stage. Differences emerge in childhood and widen over time, coinciding with the development of gender identity (at age 3-9 years), sexual attraction (at age 10-13 years), and racial and ethnic minority stressors, which may collectively increase mental health challenges among SGD youths.^7,10,52,53^ Furthermore, diagnostic codes showed similar age-related patterns, shifting from primarily mental disorders in early childhood (eg, disturbances of conduct or emotion, attention-deficit disorders, etc) to anxiety-related disorders in young adulthood. Although disorder-specific trajectories were not modeled, this pattern is consistent with research suggesting that early behavioral or emotional problems may evolve into a range of anxiety and mood disorders in adulthood.^54,55,56^

SGD youths were nearly twice as likely to visit the emergency department and had higher inpatient service use compared with their cisgender heterosexual peers, suggesting that, despite their higher engagement with outpatient services throughout development, mental health crises may still escalate to the point of requiring emergency or inpatient care. These patterns likely reflect both the severity of mental health problems among SGD youths^7,57^ as well as important deficiencies in available outpatient services.^58,59,60^ This aligns with previous research using Quebec administrative data,^61^ showing that frequent emergency service users, compared with less frequent users, have more severe mental health problems and a higher risk of hospitalization, despite greater outpatient service use. Among SGD youths, higher emergency service use alongside greater outpatient engagement^60^ has been interpreted as an indicator of unmet mental health needs and may reflect pervasive inequities in access to appropriate mental health care.^62,63,64,65,66,67^

SGD youths assigned female were more likely than cisgender heterosexual females to use emergency and inpatient services, whereas comparisons among those assigned male were not interpretable due to small cell sizes, consistent with evidence that females are more likely to identify as sexually diverse.^68,69^ Notably, among youths assigned female, outpatient service use increased over time in both SGD and cisgender heterosexual youths, with comparable patterns of increase between groups. Among youths assigned male, outpatient service use appeared to increase only among SGD youths, although trajectories were also similar between groups. Existing evidence is mixed regarding whether mental health trajectories among SGD youth differ by sex assigned at birth,^24,70,71^ highlighting the need for further research, particularly among SGD youths assigned male.

### Limitations

Several limitations of this study should be noted. First, reliance on linked administrative health records restricted analyses to physician services covered under Quebec’s provincial health insurance program. Services not billed through this system, including psychotherapy, are not captured,^20^ likely leading to an underestimation of mental health service use. Second, observed service use differences should not be interpreted solely as differences in underlying mental health need, as they may also reflect variation in help-seeking pathways, including parent-initiated access to care in childhood and parental recognition of mental health needs.^72,73,74^

Third, 38.1% of participants were lost to follow-up by adulthood and lacked SGD data, consistent with other cohorts.^75,76^ Although survey weights were applied, selection bias remains possible if missingness was related to SGD status; however, there is no evidence that SGD individuals are less likely to participate in surveys than cisgender heterosexual individuals.^77,78,79,80^

Fourth, although service use was examined over time, SGD status was assessed only at age 23 years, precluding analyses of how the initial realization, disclosure, timing, or fluidity of SGD identity relates to mental health service use trajectories.^81,82,83^ Fifth, we did not account for key risk and protective factors that could influence mental health service use among SGD youths. Discrimination and bullying contribute to disparities,^52,53^ while social support may buffer these effects.^7,84^ Earlier disclosure of sexual or gender identity may also increase minority stress.^85^

Sixth, the small number of gender diverse youths—about half of whom also identified as sexually diverse—required combining sexual and gender diversity in analyses, precluding examination of service use patterns specific to gender diverse youths, who may experience earlier and more pronounced mental health challenges.^85,86^ Furthermore, limited statistical power prevented disaggregation of service use patterns by sexual orientation.^35,87,88^ Notably, sexually diverse females in our sample mainly identified as bisexual or mostly heterosexual, identities associated with elevated mental health problems.^13,14,15,35,89^

Finally, inpatient service use was also rare (72 participants [5.4%]), limiting statistical power. Power was also reduced in sex-stratified analyses, as only 92 of 340 SGD youths (27.1%) were assigned male compared with 248 of 340 (72.9%) assigned female.

## Conclusions

This analysis of a large population-based cohort from Quebec showed that SGD youths were more likely than their cisgender heterosexual peers to use mental health services. These disparities emerged in childhood and widened through young adulthood, highlighting the importance of early preventative efforts—such as reducing bullying and fostering supportive environments—to mitigate mental health risks among SGD youths. Their reliance on emergency services suggests that existing supports may be insufficient, highlighting the need for accessible, affirming, and culturally competent care.

## Notes

Supplement 1. **eTable 1.** Comparison of Early Life Characteristics Between Included (n = 1326) and Excluded (n = 794) Participants **eTable 2.** Classification of Sexual Orientation Using an Adapted Klein Sexual Orientation Grid (KSOG) **eTable 3.** Administrative Data Sources and Diagnostic Codes for Mental Health Service Use by Service Type **eTable 4.** Sexual Orientation and Gender Identity Characteristics of the Study Sample (n = 1326), Weighted **eTable 5.** Prevalence of Any Mental Health Service Use by Service Type Among Cisgender Heterosexual and Sexually and Gender Diverse Youth (n = 1326); Weighted **eTable 6.** Prevalence of Any Outpatient Service Use by Diagnostic Category (n = 1326); Weighted **eTable 7.** Prevalence of Any Emergency Service Use by Reason for Visit Codes (n = 1326); Weighted **eTable 8.** Prevalence of Any Inpatient Service Use by Diagnostic Category (n = 1326); Weighted **eTable 9.** Maximum Likelihood Robust (MLR) and Weighted Least Squares With Mean and Variance Adjustment (WLSMV) Fit Indices **eTable 10.** Growth Curve Modelling of Mental Health Service Use from 1 to 23 Years Among Sexually and Gender Diverse Youth Compared to Cisgender Heterosexual Youth (n = 1326) With WLSMV; Weighted **eTable 11.** Prevalence of Emergency and Inpatient Mental Health Service Use in Cisgender Heterosexual Versus Sexually and Gender Diverse Assigned Males (n = 635); Weighted **eFigure 1.** Association Between Sexually and Gender Diversity and Any Outpatient Mental Health Service Use by Developmental Period (n = 1326); Weighted **eFigure 2.** Association Between Sexual and Gender Diversity and Any Mental Health Service Use by Service Type Among Assigned Females (n = 691) and Assigned Males (n = 635); Weighted **eFigure 3.** Outpatient Mental Health Service Use Trajectories by Sexual and Gender Diversity and Sex Assigned at Birth Among Assigned Females (n = 691) and Assigned Males (n = 635); Weighted
eReferences

Supplement 2.
Data Sharing Statement
