# Supplementary material for: Mental Health Service Use in Sexually and Gender Diverse Youths and Cisgender Heterosexual Youths
Source: JAMA Netw Open. 2026 Aug 14;9(8):e2626797. doi: 10.1001/jamanetworkopen.2026.26797 (PMC13476833; doi:10.1001/jamanetworkopen.2026.26797)
Supplement: Supplementary file 1 [file jamanetwopen-e2626797-s001.pdf]

Supplementary Online Content

Anzarouth L, MacNeil S, Ryan NC, et al. Mental health service use in sexually and gender diverse youths and cisgender heterosexual youths. *JAMA Netw Open or Health Forum.* 2026;9(8):e2626797. doi:10.1001/jamanetworkopen.2026.26797

eTable 1. Comparison of Early Life Characteristics Between Included (n = 1326) and Excluded (n = 794) Participants

eTable 2. Classification of Sexual Orientation Using an Adapted Klein Sexual Orientation Grid (KSOG)

eTable 3. Administrative Data Sources and Diagnostic Codes for Mental Health Service Use by Service Type

eTable 4. Sexual Orientation and Gender Identity Characteristics of the Study Sample (n = 1326), Weighted

eTable 5. Prevalence of Any Mental Health Service Use by Service Type Among Cisgender Heterosexual and Sexually and Gender Diverse Youth (n = 1326); Weighted

eTable 6. Prevalence of Any Outpatient Service Use by Diagnostic Category (n = 1326); Weighted

eTable 7. Prevalence of Any Emergency Service Use by Reason for Visit Codes (n = 1326); Weighted

eTable 8. Prevalence of Any Inpatient Service Use by Diagnostic Category (n = 1326); Weighted

eTable 9. Maximum Likelihood Robust (MLR) and Weighted Least Squares With Mean and Variance Adjustment (WLSMV) Fit Indices

eTable 10. Growth Curve Modelling of Mental Health Service Use from 1 to 23 Years Among Sexually and Gender Diverse Youth Compared to Cisgender Heterosexual Youth (n = 1326) With WLSMV; Weighted

eTable 11. Prevalence of Emergency and Inpatient Mental Health Service Use in Cisgender Heterosexual Versus Sexually and Gender Diverse Assigned Males (n = 635); Weighted

eFigure 1. Association Between Sexually and Gender Diversity and Any Outpatient Mental Health Service Use by Developmental Period (n = 1326); Weighted

eFigure 2. Association Between Sexual and Gender Diversity and Any Mental Health Service Use by Service Type Among Assigned Females (n = 691) and Assigned Males (n = 635); Weighted

© 2026 Anzarouth L et al. *JAMA Network Open*.

eFigure 3. Outpatient Mental Health Service Use Trajectories by Sexual and Gender Diversity and Sex Assigned at Birth Among Assigned Females (n = 691) and Assigned Males (n = 635); Weighted

eReferences

This supplementary material has been provided by the authors to give readers additional information about their work.

© 2026 Anzarouth L et al. *JAMA Network Open*.

eTable 1. Comparison of Early Life Characteristics Between Included (n = 1326) and Excluded (n = 794) Participants

	**Included N=1326**	**Excluded N=794**	**Effect Size**
	N (%)	N (%)	Cramer's V[e]
Sex			**0.22**
Male	562 (42.4)	518 (65.2)	
Female	764 (57.6)	276 (34.8)	
Race and ethnicity[a]			**0.10**
Non-White	107 (8.1)	112 (14.1)	
White	1216 (91.9)	682 (85.9)	
Sufficient household income[b]			**0.15**
Yes	1069 (81.5)	529 (68.7)	
No	242 (18.5)	241 (31.3)	
Single parent household			**0.07**
Yes	88 (6.7)	83 (10.5)	
No	1235 (93.3)	706 (89.5)	
Maternal depressive symptoms[c]			**0.08**
Yes	194 (14.7)	163 (20.7)	
No	1129 (85.3)	626 (79.3)	
	M(SD)	M(SD)	Spearman Correlation[e]
Maternal age in years at child's birth	29.2 (5.10)	28.4 (5.39)	**0.07**
Internalizing symptoms[d]	0.89 (1.20)	0.92 (1.26)	0.004
Externalizing symptoms[d]	4.99 (2.90)	5.16 (3.10)	0.02

Note: Data compiled from the Québec Longitudinal Study of Child Development (1998–2021), © Gouvernement du Québec, Institut de la statistique du Québec[1].
All measures are reported by parents when children were 5 months old, unless otherwise indicated.
Bolded values are statistically significant at $p < .05$.
[a] Defined as parental identification of the children to any of 11 non-White ethnic and racial categories, including Arab/West Asian, Black, Chinese, Filipino, Japanese, Korean, Latin-American, Native/Aboriginal People South Asian, South East Asian, or other race or ethnicity.
[b] Insufficiency defined as spending more than 20% of the annual income for basic needs, in addition to the average proportion spent by households of similar size and regional population density.
[c] Based on the 12-item version of the Center for Epidemiologic Studies–Depression scale, rescaled to range from 0 to 10, with higher scores indicating more depressive symptoms[2]
[d] Based on the Behavior Questionnaire scores (range, 1-3) of 6 items for internalizing and 10 items for externalizing behaviors, with higher scores indicating more internalizing/externalizing symptoms.[3] Measured when children were 29 months of age; missing values were replaced with data collected at 17 months.
[e] Effect sizes for comparing included and excluded participants are Cramer V and absolute Spearman correlations (range, 0.000-1.000

© 2026 Anzarouth L et al. *JAMA Network Open*.

eTable 2. Classification of Sexual Orientation Using an Adapted Klein Sexual Orientation Grid (KSOG)

Survey item: *"People experience different forms of sexual attraction. How do you identify yourself?"*

Original Response Option	Descriptive Category	Grouping Used in Main Analyses
Exclusively heterosexual	Heterosexual	Heterosexual
Predominantly heterosexual, only incidentally homosexual	Mostly heterosexual	Sexually diverse
Predominantly heterosexual, but more than incidentally homosexual	Mostly heterosexual	Sexually diverse
Bisexual	Bisexual	Sexually diverse
Predominantly homosexual, but more than incidentally heterosexual	Mostly lesbian/gay	Sexually diverse
Predominantly homosexual, only incidentally heterosexual	Mostly lesbian/gay	Sexually diverse
Exclusively homosexual	Lesbian/gay	Sexually diverse
Asexual	Unsure/questioning or asexual	Sexually diverse
Questioning/unsure	Unsure/questioning or asexual	Sexually diverse

Note: Data compiled from the Québec Longitudinal Study of Child Development (1998–2021), © Gouvernement du Québec, Institut de la statistique du Québec[1].
Klein F, Sepekoff B, Wolf TJ. Sexual orientation: a multi-variable dynamic process. *J Homosex*. 1985;11(1-2):35-49. doi:10.1300/J082v11n01_04[4]

© 2026 Anzarouth L et al. *JAMA Network Open*.

eTable 3. Administrative Data Sources and Diagnostic Codes for Mental Health Service Use by Service Type

Service Type	Data Source	Time Period	Age Range	Diagnostic Codes
**Outpatient Services**	Régie de l'assurance maladie du Québec (RAMQ)	1997–2021[a]	1–23 years	1997–2021:<br>**ICD-9:** 290–319, E950-E959<br>2019–2021:<br>**ICD-10:** F00–F99, X60-X84, Y87.0
**Inpatient Services** (i.e., Hospitalizations)	Maintenance et exploitation des données pour l'étude de la clientèle hospitalière (MED-ÉCHO)	2006–2021[b]	8–23 years	**ICD-10:** F00–F99, X60-X84, Y87.0
**Emergency Department**	Banque de données communes des urgences (BDCU)	2016–2021[c]	18–23 years	**Reason for Visit codes:** 0680–0681, 0600, 0601, 0602, 0603, 1300, 0400

[a] From 1997 to 2018, diagnoses were coded using **ICD-9** (ages 1–18). Beginning in 2019, **ICD-10** codes were introduced, and both ICD-9 and ICD-10 were used in parallel from 2019 to 2021 (ages 18–23).
[b] From 1997 to 2006, diagnoses were coded using **ICD-9**, while **ICD-10** was used from 2006 to 2021. We extracted inpatient diagnoses using ICD-10 from 2006 onward (ages 8-23), as hospitalizations before this age were rare.
[c] To capture emergency department visits during adulthood (ages 18–23), we restricted analyses to 2016–2021, since the BDCU registry was established only in 2014 (age 16).
[a-c] As reported in: Wilchesky, M., & Suissa, S. (2021). The Régie de l'assurance maladie du Québec (RAMQ) Databases. In M. Sturkenboom & T. Schink (Eds.), *Databases for Pharmacoepidemiological Research. Springer Series on Epidemiology and Public Health.* Cham: Springer[5].

© 2026 Anzarouth L et al. *JAMA Network Open*.

eTable 4. Sexual Orientation and Gender Identity Characteristics of the Study Sample (n = 1326), Weighted

	**Total (N=1326)**		**Assigned Female (N=691)**		**Assigned Male (N=635)**	
	**N**	**%**	**N**	**%**	**N**	**%**
Cisgender and heterosexual	986	74.4	443	64.1	543	85.5
Sexually and gender diverse	340	25.6	248	35.9	92	14.5
Sexual Orientation[a]						
Sexually diverse	324	24.5	242	35.0	82	12.9
Mostly heterosexual	156	11.8	124	17.9	32	5.0
Bisexual	85	6.5	71	10.3	15	2.4
Mostly lesbian/gay	19	1.4	10	1.4	9	1.4
Lesbian/gay	27	2.0	9	1.3	18	2.8
Unsure/questioning or asexual	37	2.8	28	4.1	9	1.4
Heterosexual	1000	75.5	449	65.0	551	86.8
Gender Identity						
Gender diverse	32	2.4	17	2.5	15	2.4
Cisgender	1294	97.6	674	97.5	620	97.6

Note: Data compiled from the Québec Longitudinal Study of Child Development (1998–2021), © Gouvernement du Québec, Institut de la statistique du Québec[1]. Data were weighted to adjust for selective attrition.
Note: Ns and percentages are weighted; totals may differ slightly due to rounding of weighted counts.
[a]Two participants identified as gender diverse but provided no information for their sexual orientation. An additional five individuals identified as asexual. Due to rules for ISQ disclosure regulations, data pertaining to these participants alone cannot be reported. An additional 14 participants identified as both heterosexual and gender diverse.

eTable 5. Prevalence of Any Mental Health Service Use by Service Type Among Cisgender Heterosexual and Sexually and Gender Diverse Youth (n = 1326); Weighted

	No. Participants (%)		
**Age**	**Cisgender Heterosexual (N=986)**	**Sexually and Gender Diverse (N=340)**	**Total (N=1326)**
Any Outpatient Services			
Overall (1–23)	504 (51.1)	203 (59.7)	707 (53.3)
Preschool (1–5)	121 (12.3)	29 (8.5)	150 (11.3)
Childhood (6–12)	209 (21.2)	83 (24.4)	292 (22.0)
Adolescence (13–17)	231 (23.4)	96 (28.2)	327 (24.7)
Young Adulthood (18–23)	302 (30.6)	139 (40.9)	440 (33.2)
Any Emergency Department Visits			
18–23	75 (7.6)	51 (15.0)	126 (9.5)
Any Inpatient Services			
8–23	47 (4.8)	25 (7.4)	72 (5.4)

Note: Data compiled from the Québec Longitudinal Study of Child Development (1998–2021), © Gouvernement du Québec, Institut de la statistique du Québec[1]. Data were weighted to adjust for selective attrition.
Note: Ns and percentages are weighted; totals may differ slightly due to rounding of weighted counts.

© 2026 Anzarouth L et al. *JAMA Network Open*.

eTable 6. Prevalence of Any Outpatient Service Use by Diagnostic Category (n = 1326); Weighted

		**No. Participants (%)** [a]			
**Category**	**ICD 9 Code(s)**	**Preschool (Ages 1-5)**	**Childhood (Ages 6-12)**	**Adolescence (Ages 13-17)**	**Young Adulthood (Ages 18-23)**
Organic psychotic disorders	290–294				16 (1.2)
Other psychotic disorders	295–299		13 (1.0)	25 (1.9)	36 (2.7)
Neurotic (anxiety-related) disorders	300	23 (1.7)	61 (4.6)	130 (9.8)	233 (17.6)
Personality disorders	301		5 (0.4)	12 (0.9)	32 (2.4)
Sexual deviations and disorders	302				
Psychoactive substance use disorders	303–305			11 (0.8)	22 (1.7)
Other (adult onset) mental disorders	306–311	62 (4.7)	96 (7.2)	130 (9.8)	194 (14.6)
Mental disorders in childhood	312–316	84 (6.3)	200 (15.1)	170 (12.8)	119 (9.0)
Mental retardation	317–319				
Suicide and self-inflicted injury	E950–E959				
**Category**	**ICD 10 Code(s)**	**Preschool (Ages 1-5)**	**Childhood (Ages 6-12)**	**Adolescence (Ages 13-17)**	**Young Adulthood (Ages 18-23)**
Organic, including symptomatic, mental disorders	F00–F09				
Mental and behavioral disorders due to use of psychoactive substances	F10–F19				

© 2026 Anzarouth L et al. *JAMA Network Open*.

Category	ICD 10 Code(s)	Preschool (Ages 1-5)	Childhood (Ages 6-12)	Adolescence (Ages 13-17)	Young Adulthood (Ages 18-23)
Schizophrenia, schizotypal, and delusional disorders	F20–F29				
Mood [affective] disorders	F30–F39				18 (1.4)
Anxiety, dissociative, stress–related, somatoform and other nonpsychotic mental disorder	F40–F49				41 (3.1)
Behavioral syndromes (e.g., eating disorders)	F50–F59				
Personality and behavior disorders in adult persons	F60–F69				
Mental retardation	F70–F79				
Disorders of psychological development	F80–F89				
Behavioral and emotional disorders with onset usually occurring in childhood and adolescence	F90–F98				5 (0.4)
Mental disorders, not specified	F99				
Intentional self-harm	X60–X84				
Late effects of intentional self-harm	Y87.0				

Note: Data compiled from the Québec Longitudinal Study of Child Development (1998–2021), © Gouvernement du Québec, Institut de la statistique du Québec. Data were weighted to adjust for selective attrition.
Shaded boxes indicate cells with fewer than 5 participants; values are not reported to protect confidentiality[1].
[a] Data were extracted from the Regie de l’assurance maladie de Québec (RAMQ) physicians claims database, pertaining to all renumerated outpatient medical services in Québec. Reported in: Wilchesky M, Suissa S. The Régie de l’assurance maladie du Québec (RAMQ) Databases. In: Sturkenboom M, Schink T, eds. *Databases for Pharmacoepidemiological Research*. Springer International Publishing; 2021:215-230. doi:10.1007/978-3-030-51455-6_18[5]

© 2026 Anzarouth L et al. *JAMA Network Open*.

eTable 7. Prevalence of Any Emergency Service Use by Reason for Visit Codes (n = 1326); Weighted

Category	Reason for Visit Code(s)	% (No. Participants) [a]
Suicidal ideation and attempt	0680-0681	49 (3.7)
Depression or auto mutilation	0600	24 (1.8)
Anxiety/situational crisis	0601	58 (4.4)
Hallucinations	0602	
Insomnia	0603	
Drug use	1300	16 (1.2)
Anorexia	0400	

Note: Data compiled from the Québec Longitudinal Study of Child Development (1998–2021), © Gouvernement du Québec, Institut de la statistique du Québec[1]. Data were weighted to adjust for selective attrition.
Shaded boxes indicate cells with fewer than 5 participants; values are not reported to protect confidentiality.
[a] Data were extracted from the Banque de données communes des urgences (BDCU) database, pertaining to all services provided in emergency departments in Québec. Reported in: Wilchesky M, Suissa S. The Régie de l'assurance maladie du Québec (RAMQ) Databases. In: Sturkenboom M, Schink T, eds. *Databases for Pharmacoepidemiological Research*. Springer International Publishing; 2021:215-230. doi:10.1007/978-3-030-51455-6_18[5]

© 2026 Anzarouth L et al. *JAMA Network Open*.

eTable 8. Prevalence of Any Inpatient Service Use by Diagnostic Category (n = 1326); Weighted

**Category**	**ICD 10 Code(s)**	**No. Participants (%)** [a]
Organic, including symptomatic, mental disorders	F00–F09	
Mental and behavioral disorders due to use of psychoactive substances	F10–F19	22 (1.7)
Schizophrenia, schizotypal, and delusional disorders	F20–F29	7 (0.5)
Mood [affective] disorders	F30–F39	18 (1.4)
Neurotic, stress-related, and somatoform disorders	F40–F49	32 (2.4)
Behavioral syndromes (e.g., eating disorders)	F50–F59	
Personality and behavior disorders in adult persons	F60–F69	13 (1.0)
Mental retardation	F70–F79	
Disorders of psychological development	F80–F89	10 (0.8)
Behavioral and emotional disorders with onset usually occurring in childhood and adolescence	F90–F98	27 (2.0)
Mental disorders, not specified	F99	
Intentional self-harm	X60–X84	
Late effects of intentional self-harm	Y87.0	

Note: Data compiled from the Québec Longitudinal Study of Child Development (1998–2021), © Gouvernement du Québec, Institut de la statistique du Québec[1]. Data were weighted to adjust for selective attrition.
Shaded boxes indicate cells with fewer than 5 participants; values are not reported to protect confidentiality.
[a] Data were extracted from the Maintenance et exploitation des données pour l'étude de la clientèle hospitalière (MED-ÉCHO) database, pertaining to all discharges from hospitals in Québec. Reported in: Wilchesky M, Suissa S. The Régie de l'assurance maladie du Québec (RAMQ) Databases. In: Sturkenboom M, Schink T, eds. *Databases for Pharmacoepidemiological Research*. Springer International Publishing; 2021:215-230. doi:10.1007/978-3-030-51455-6_18[5]

© 2026 Anzarouth L et al. *JAMA Network Open*.

eTable 9. Maximum Likelihood Robust (MLR) and Weighted Least Squares With Mean and Variance Adjustment (WLSMV) Fit Indices

Estimation Method	Model	Global χ² Fit	RMSEA (90% CI, p-close)	CFI/TLI	SRMR
**MLR**	Unconditional	$\chi^2(10)$ = 50.16 *P*=<.001	–	–	–
**WLSMV**	Unconditional	$\chi^2(2)$ = 1.62, *P*=.45	0.00(0.00–0.05), p=0.94	CFI=1.00, TLI=1.00	0.01
	Unadjusted	$\chi^2(4)$ = 5.91, P=.21	0.02 (0.00–0.05), p=0.96	CFI=0.996, TLI=0.990	0.02
	Adjusted for SES only	$\chi^2(6)$ = 13.34, *P*=.04	0.03 (0.01–0.05), p=0.92	CFI=0.985, TLI=0.965	0.02
	Adjusted for SES, Assigned Females	$\chi^2(6)$ = 6.42, *P*=.38	0.01 (0.00–0.05), p=0.96	CFI=0.998, TLI=0.996	0.02

Note: Data compiled from the Québec Longitudinal Study of Child Development (1998–2021), © Gouvernement du Québec, Institut de la statistique du Québec[1].

© 2026 Anzarouth L et al. *JAMA Network Open*.

eTable 10. Growth Curve Modelling of Mental Health Service Use from 1 to 23 Years Among Sexually and Gender Diverse Youth Compared to Cisgender Heterosexual Youth (n = 1326) With WLSMV; Weighted

	**Intercept**				**Slope**			
	**b (SE)**	**95% C.I.**		**P-Value**	**b (SE)**	**95% C.I.**		**P-Value**
		**Lower**	**Upper**			**Lower**	**Upper**	
Unconditional model (N=1326)	[Reference]	NA	NA	NA	-0.65 (0.41)	-1.45	0.15	.11
Sexually and gender diverse (v. cisgender heterosexual) (Unadjusted, N=1326)	-0.22 (0.11)	-0.44	0.003	.05	0.51 (0.20)	0.12	0.89	.01
Sexually and gender diverse	-0.22 (0.11)	-0.44	0.003	.05	-0.30 (0.34)	-0.97	0.37	.38
Cisgender heterosexual	[Reference]	NA	NA	NA	-0.81 (0.46)	-1.70	0.09	.08
Sexually and gender diverse (v. cisgender heterosexual) (Adjusted for SES only, N=1320)	-0.20 (0.11)	-0.42	0.03	.09	0.52 (2.11)	0.11	0.94	.01
Sexually and gender diverse	-0.20 (0.11)	-0.42	0.03	.09	-0.37 (0.37)	-1.10	0.36	.32
Cisgender heterosexual	0.00 (0.00)	-0.001	0.001	0.52	-0.89 (0.50)	-1.88	0.10	.08
Sexually and gender diverse (v. cisgender heterosexual) (Adjusted for SES, Assigned females, N=688)	-0.10 (0.13)	-0.36	0.16	.47	0.21 (0.12)	-0.01	0.44	.06
Sexually and gender diverse	-0.10 (0.13)	-0.36	0.16	.47	0.20 (0.13)	-0.05	0.45	.11
Cisgender heterosexual	0.00 (0.00)	-0.001	0.001	0.83	-0.01 (0.17)	-0.34	0.31	.95

Note: Data compiled from the Québec Longitudinal Study of Child Development (1998–2021), © Gouvernement du Québec, Institut de la statistique du Québec[1]. Data were weighted to adjust for selective attrition.
Note: Unconditional growth intercept is fixed to zero.
Note: Estimates are presented on the *probit* scale, explaining the negative intercepts.
Model parameters are estimated for the indicated variable (coded 1) in reference to the condition in parentheses (coded 0).
SE = Standard Error.

© 2026 Anzarouth L et al. *JAMA Network Open*.

eTable 11. Prevalence of Emergency and Inpatient Mental Health Service Use in Cisgender Heterosexual Versus Sexually and Gender Diverse Assigned Males (n = 635); Weighted

	**No. Participants (%)** [b]	
**Service Type**	**Cisgender Heterosexual Males (N=543)**	**Sexually and Gender diverse Assigned Males (N=92)**
Emergency	34 (6.3)	10 (10.9)
Inpatient	27 (5.0)	

Note: Data compiled from the Québec Longitudinal Study of Child Development (1998–2021), © Gouvernement du Québec, Institut de la statistique du Québec[1]. Data were weighted to adjust for selective attrition.
Shaded boxes indicate cells with fewer than 5 participants; values are not reported to protect confidentiality.
Reference group: Cisgender and heterosexual males.
[a] Estimates are based on odds ratios from logistic regressions with 95% confidence intervals.
[b] Data were extracted from the the Banque de données communes des urgences (BDCU) and Maintenance et exploitation des données pour l'étude de la clientèle hospitalière (MED-ÉCHO) databases. Reported in: Wilchesky M, Suissa S. The Régie de l'assurance maladie du Québec (RAMQ) Databases. In: Sturkenboom M, Schink T, eds. *Databases for Pharmacoepidemiological Research*. Springer International Publishing; 2021:215-230. doi:10.1007/978-3-030-51455-6_18[5]

eFigure 1. Association Between Sexually and Gender Diversity and Any Outpatient Mental Health Service Use by Developmental Period (n = 1326); Weighted[a]

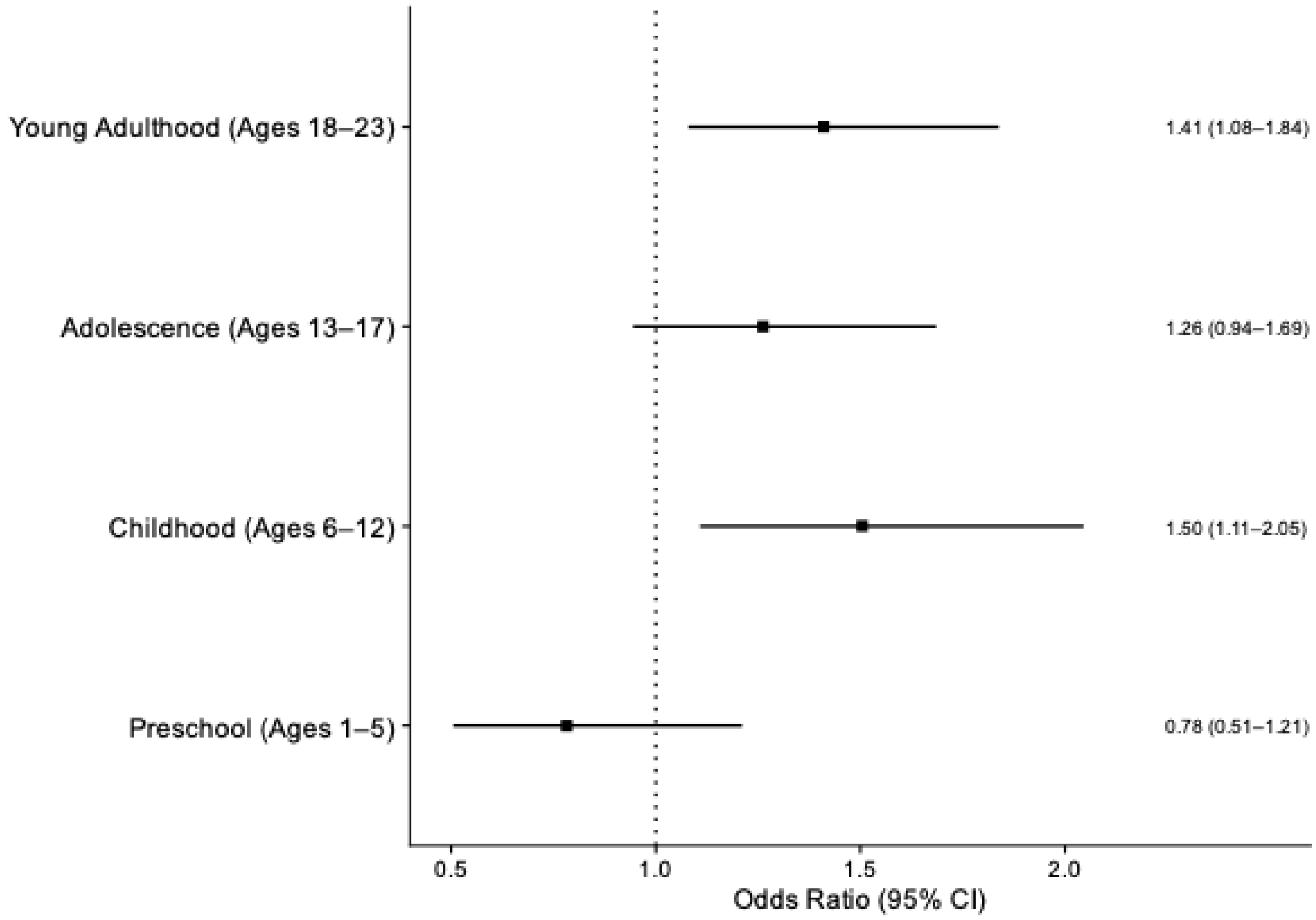

Note: Data compiled from the Québec Longitudinal Study of Child Development (1998–2021), © Gouvernement du Québec, Institut de la statistique du Québec[1]. Data were weighted to adjust for selective attrition.
OR = Odds Ratio; CI = Confidence Interval.
Reference group: Cisgender and Heterosexual youth.
[a] Estimates are based on odds ratios from logistic regressions with 95% CI.

eFigure 2. Association Between Sexual and Gender Diversity and Any Mental Health Service Use by Service Type Among Assigned Females (n = 691) and Assigned Males (n = 635); Weighted

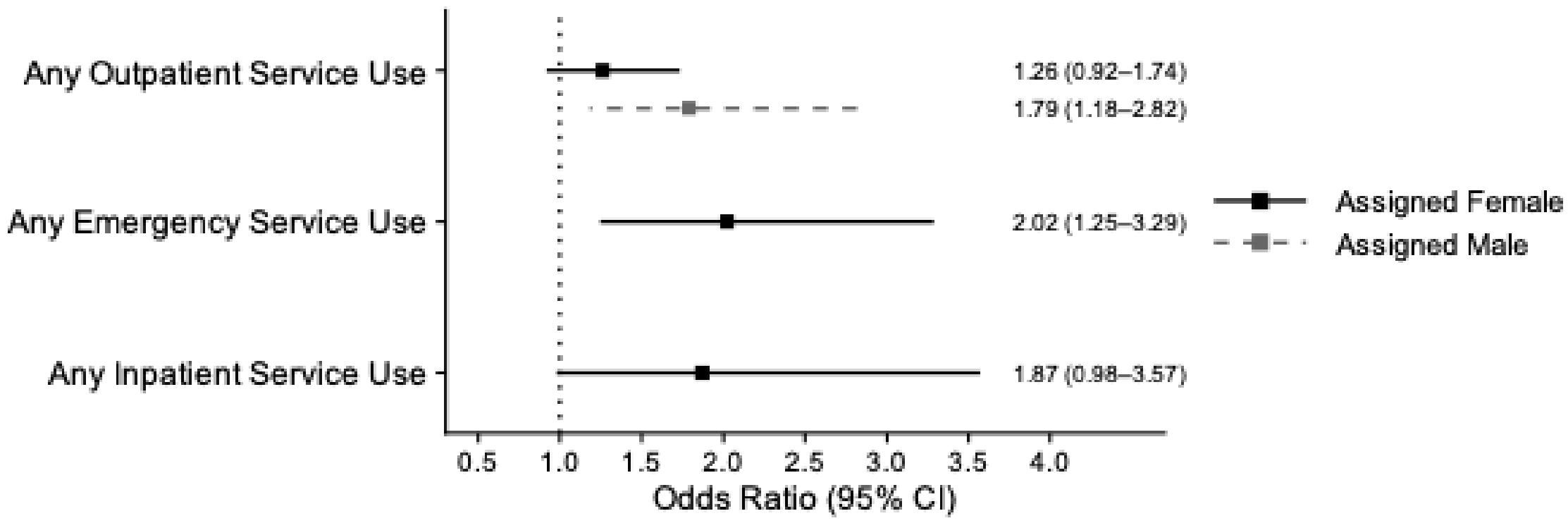

Note: Data compiled from the Québec Longitudinal Study of Child Development (1998–2021), © Gouvernement du Québec, Institut de la statistique du Québec[1]. Data were weighted to adjust for selective attrition.
OR = Odds Ratio; CI = Confidence Interval.
Reference group: Cisgender and heterosexual females.
[a] Estimates are based on odds ratios from logistic regressions with 95% CI.

© 2026 Anzarouth L et al. *JAMA Network Open*.

eFigure 3. Outpatient Mental Health Service Use Trajectories by Sexual and Gender Diversity and Sex Assigned at Birth Among Assigned Females (n = 691) and Assigned Males (n = 635); Weighted

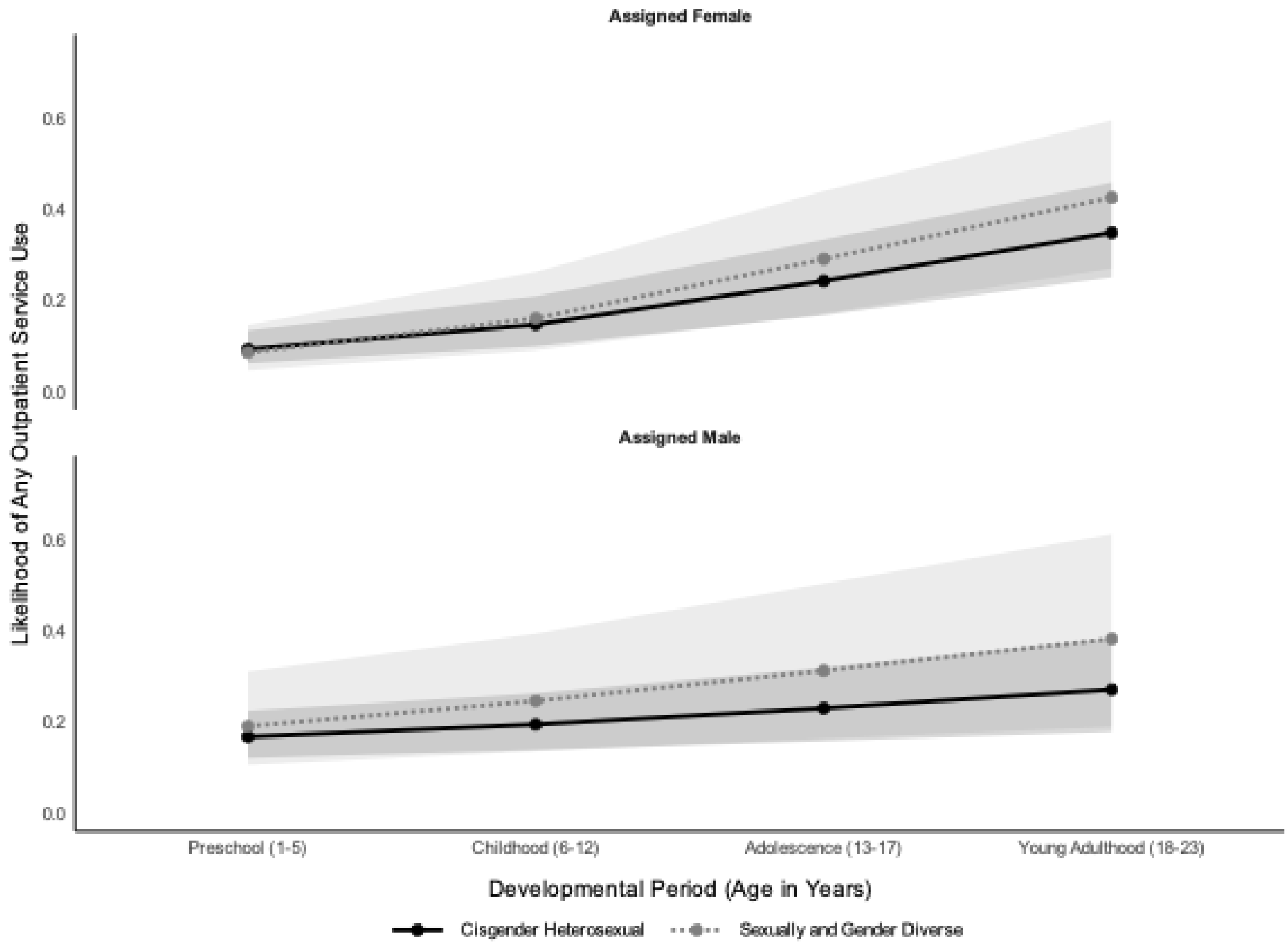

Note: Data compiled from the Québec Longitudinal Study of Child Development (1998–2021), © Gouvernement du Québec, Institut de la statistique du Québec[1]. Data were weighted to adjust for selective attrition.

eReferences

1. Institut de la statistique du Québec. Québec Longitudinal Study of Child Development (QLSCD), 1998–2021. © Gouvernement du Québec. https://www.jesuisjeserai.stat.gouv.qc.ca/

2. Radloff LS. The CES-D Scale: A self-report depression scale for research in the general population. *Appl Psychol Meas*. 1977;1(3):385-401. doi:10.1177/014662167700100306

3. Collet OA, Orri M, Tremblay RE, Boivin M, Côté SM. Psychometric properties of the social behavior questionnaire (SBQ) in a longitudinal population-based sample. *Int J Behav Dev*. 2023;47(2):180-189. doi:10.1177/01650254221113472

4. Klein F, Sepekoff B, Wolf TJ. Sexual orientation: a multi-variable dynamic process. *J Homosex*. 1985;11(1-2):35-49. doi:10.1300/J082v11n01_04

5. Wilchesky M, Suissa S. The Régie de l'assurance maladie du Québec (RAMQ) Databases. In: Sturkenboom M, Schink T, eds. *Databases for Pharmacoepidemiological Research*. Springer International Publishing; 2021:215-230. doi:10.1007/978-3-030-51455-6_18
