# Supplementary material for: Mental Health Service Use in Sexually and Gender Diverse Youths and Cisgender Heterosexual Youths
Source: JAMA Netw Open. 2026 Aug 14;9(8):e2626797. doi: 10.1001/jamanetworkopen.2026.26797 (PMC13476833; doi:10.1001/jamanetworkopen.2026.26797)
Supplement: Supplementary file 2 [file jamanetwopen-e2626797-s002.pdf]

Data Sharing Statement

Anzarouth. Mental Health Service Use in Sexually and Gender Diverse Youths and Cisgender Heterosexual Youths. *JAMA Netw Open*. Published August 03, 2026.
doi:10.1001/jamanetworkopen.2026.26797

Data
Data available: No
